# Supplementary material for: Development and Analysis of Silver Nitroprusside Nanoparticle-Incorporated Sodium Alginate Films for Banana Browning Prevention
Source: Nanomaterials (Basel). 2024 Jan 31;14(3):292. doi: 10.3390/nano14030292 (PMC10856574; doi:10.3390/nano14030292)
Supplement: Supplementary file 1 [file nanomaterials-14-00292-s001.zip › nanomaterials-2804789-supplementary.pdf]

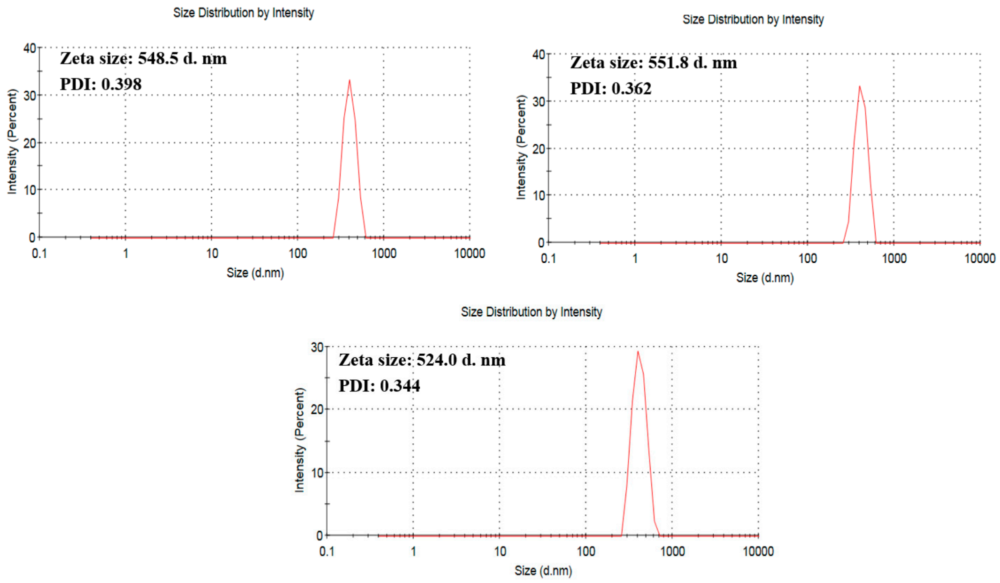

Figure S1. Size distribution and PDI values of AgNNPs.

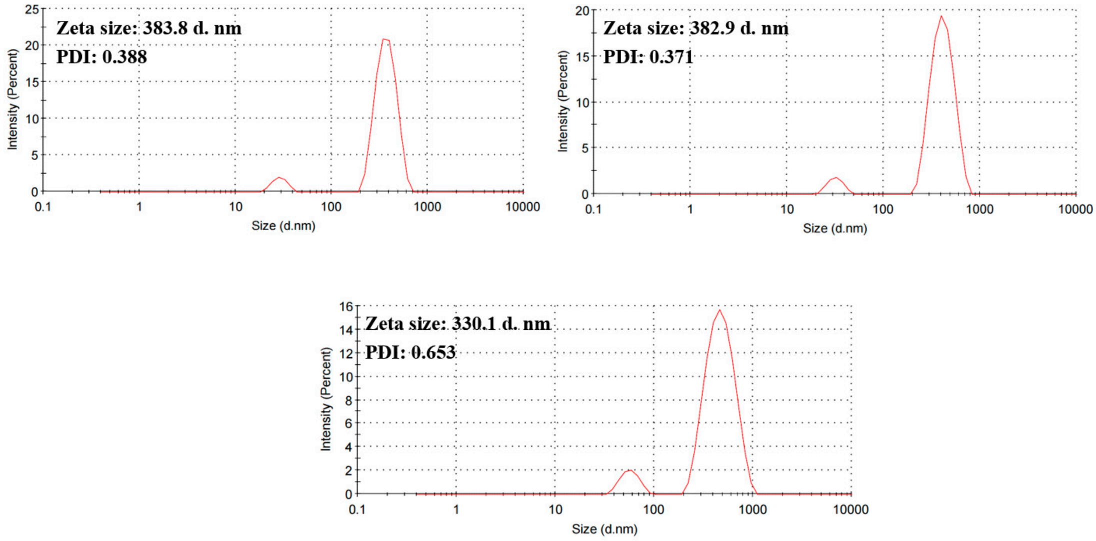

Figure S2. Size distribution and PDI values of SA-AgNNPs.

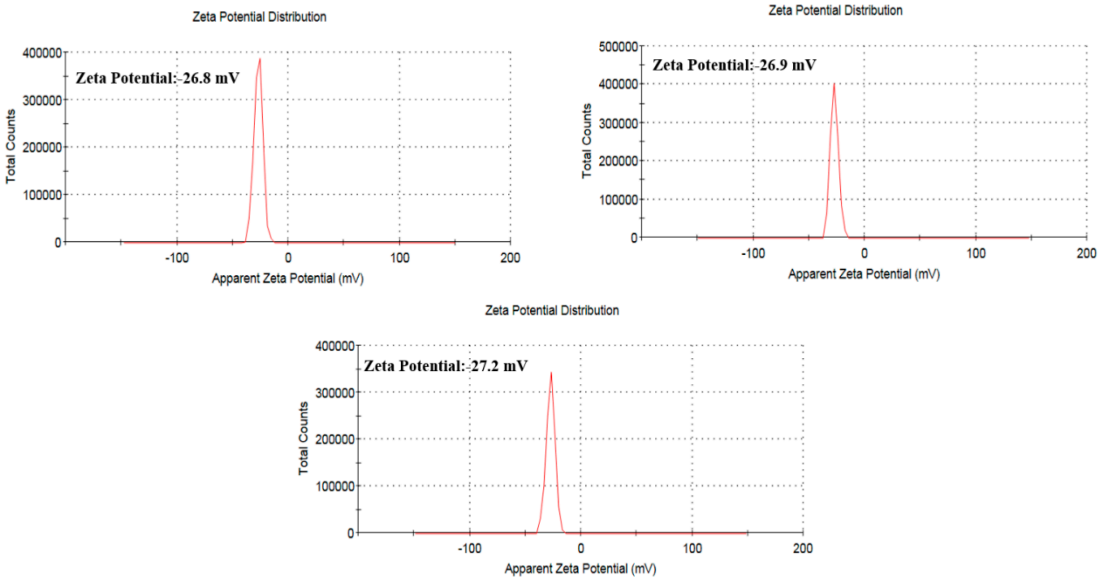

Figure S3. Zeta potentials of AgNNPs.

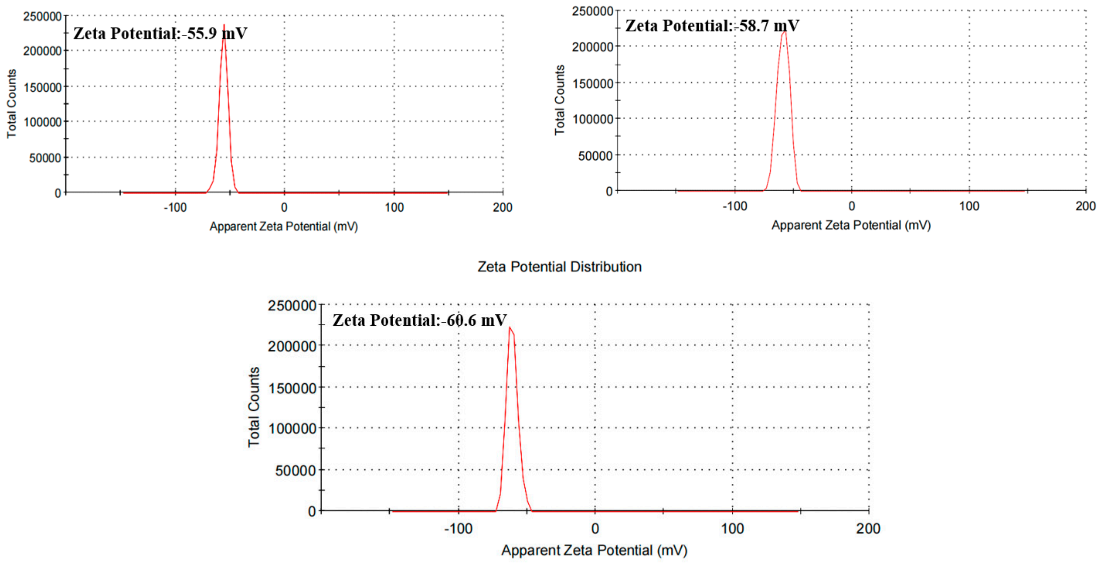

Figure S4. Zeta potentials of SA-AgNNPs.

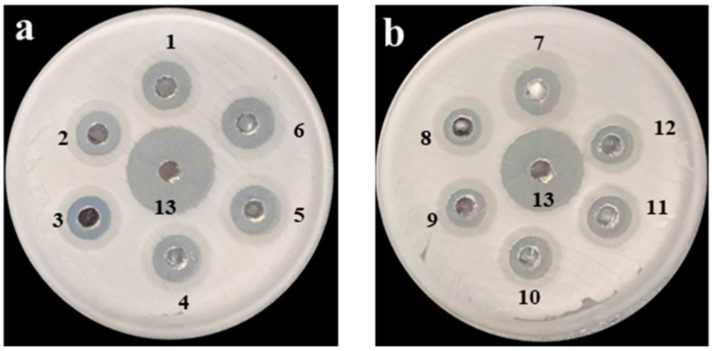

Figure S5. Antibacterial activity of SA-AgNNPs against *B. cereus* in a well diffusion assay. a: 1-2% SA, $AgNO_3$:SNP (2:1), 2-2% SA, $AgNO_3$:SNP (1:1), 3-2% SA, $AgNO_3$:SNP (1:2), 4-1% SA, $AgNO_3$:SNP (2:1), 5-

1% SA, $AgNO_3$:SNP (1:1), 6-1% SA, $AgNO_3$:SNP(1:2), b: 7-0.5% SA, $AgNO_3$:SNP (2:1), 8-0.5% SA, $AgNO_3$:SNP (1:1), 9-0.5% SA, $AgNO_3$:SNP (1:2), 10-0.25% SA, $AgNO_3$:SNP (2:1), 11-0.25% SA, $AgNO_3$:SNP (1:1), 12-0.25% SA, $AgNO_3$:SNP (1:2), 13-TCH. The final concentrations were all 50 μg.

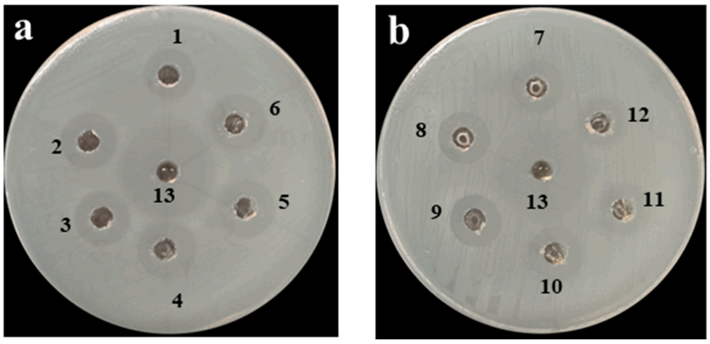

Figure S6. Antibacterial activity of SA-AgNNPs against *S. aureus* in a well diffusion assay. a: 1-2% SA, $AgNO_3$:SNP (2:1), 2-2% SA, $AgNO_3$:SNP (1:1), 3-2% SA, $AgNO_3$:SNP (1:2), 4-1% SA, $AgNO_3$:SNP (2:1), 5-1% SA, $AgNO_3$:SNP (1:1), 6-1% SA, $AgNO_3$:SNP(1:2), b: 7-0.5% SA, $AgNO_3$:SNP (2:1), 8-0.5% SA, $AgNO_3$:SNP (1:1), 9-0.5% SA, $AgNO_3$:SNP (1:2), 10-0.25% SA, $AgNO_3$:SNP (2:1), 11-0.25% SA, $AgNO_3$:SNP (1:1), 12-0.25% SA, $AgNO_3$:SNP (1:2), 13-TCH. The final concentrations were all 50 μg.

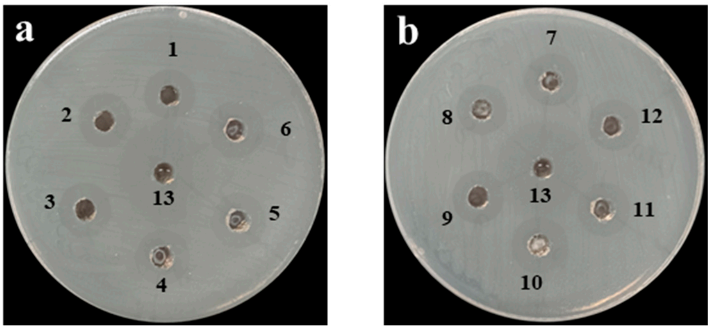

Figure S7. Antibacterial activity of SA-AgNNPs against *L. monocytogenes* in a well diffusion assay. a: 1-2% SA, $AgNO_3$:SNP (2:1), 2-2% SA, $AgNO_3$:SNP (1:1), 3-2% SA, $AgNO_3$:SNP (1:2), 4-1% SA, $AgNO_3$:SNP (2:1), 5-1% SA, $AgNO_3$:SNP (1:1), 6-1% SA, $AgNO_3$:SNP(1:2), b: 7-0.5% SA, $AgNO_3$:SNP (2:1), 8-0.5% SA, $AgNO_3$:SNP (1:1), 9-0.5% SA, $AgNO_3$:SNP (1:2), 10-0.25% SA, $AgNO_3$:SNP (2:1), 11-0.25% SA, $AgNO_3$:SNP (1:1), 12-0.25% SA, $AgNO_3$:SNP (1:2), 13-TCH. The final concentrations were all 50 μg.

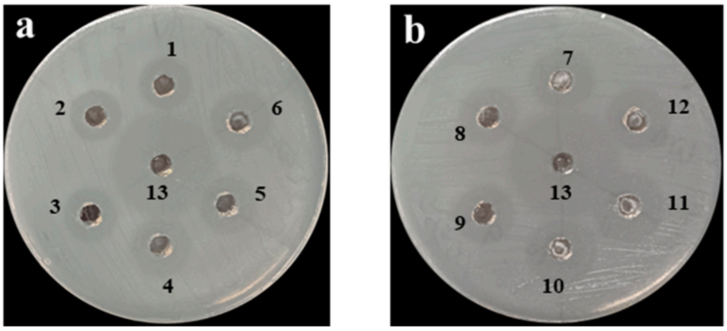

Figure S8. Antibacterial activity of SA-AgNNPs against *S. enterica* in a well diffusion assay. a: 1-2% SA, $AgNO_3$:SNP (2:1), 2-2% SA, $AgNO_3$:SNP (1:1), 3-2% SA, $AgNO_3$:SNP (1:2), 4-1% SA, $AgNO_3$:SNP (2:1), 5-1% SA, $AgNO_3$:SNP (1:1), 6-1% SA, $AgNO_3$:SNP(1:2), b: 7-0.5% SA, $AgNO_3$:SNP (2:1), 8-0.5% SA,

$AgNO_3$:SNP (1:1), 9-0.5% SA, $AgNO_3$:SNP (1:2), 10-0.25% SA, $AgNO_3$:SNP (2:1), 11-0.25% SA, $AgNO_3$:SNP (1:1), 12-0.25% SA, $AgNO_3$:SNP (1:2), 13-TCH. The final concentrations were all 50 μg.

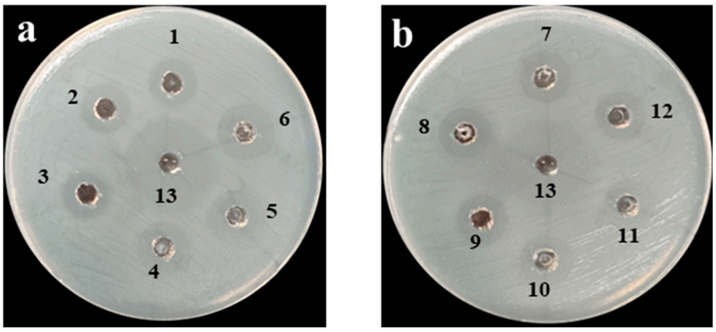

Figure S9. Antibacterial activity of SA-AgNNPs against *E. coli* in a well diffusion assay. a: 1-2% SA, $AgNO_3$:SNP (2:1), 2-2% SA, $AgNO_3$:SNP (1:1), 3-2% SA, $AgNO_3$:SNP (1:2), 4-1% SA, $AgNO_3$:SNP (2:1), 5-1% SA, $AgNO_3$:SNP (1:1), 6-1% SA, $AgNO_3$:SNP(1:2), b: 7-0.5% SA, $AgNO_3$:SNP (2:1), 8-0.5% SA, $AgNO_3$:SNP (1:1), 9-0.5% SA, $AgNO_3$:SNP (1:2), 10-0.25% SA, $AgNO_3$:SNP (2:1), 11-0.25% SA, $AgNO_3$:SNP (1:1), 12-0.25% SA, $AgNO_3$:SNP (1:2), 13-TCH. The final concentrations were all 50 μg.

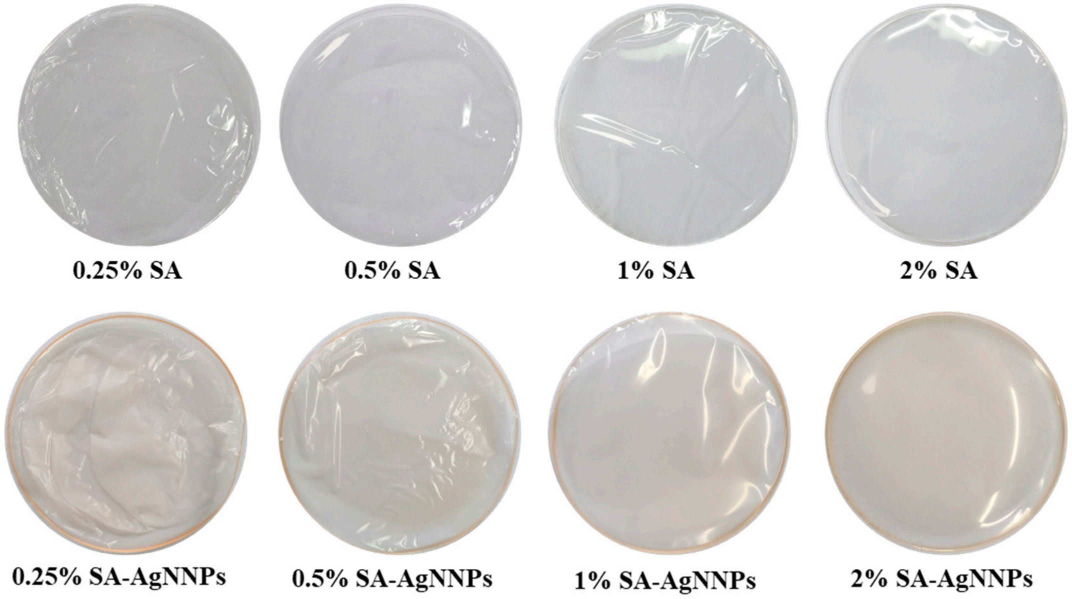

Figure S10. Digital photos of different concentrations of SA films and SA-AgNNPs films.

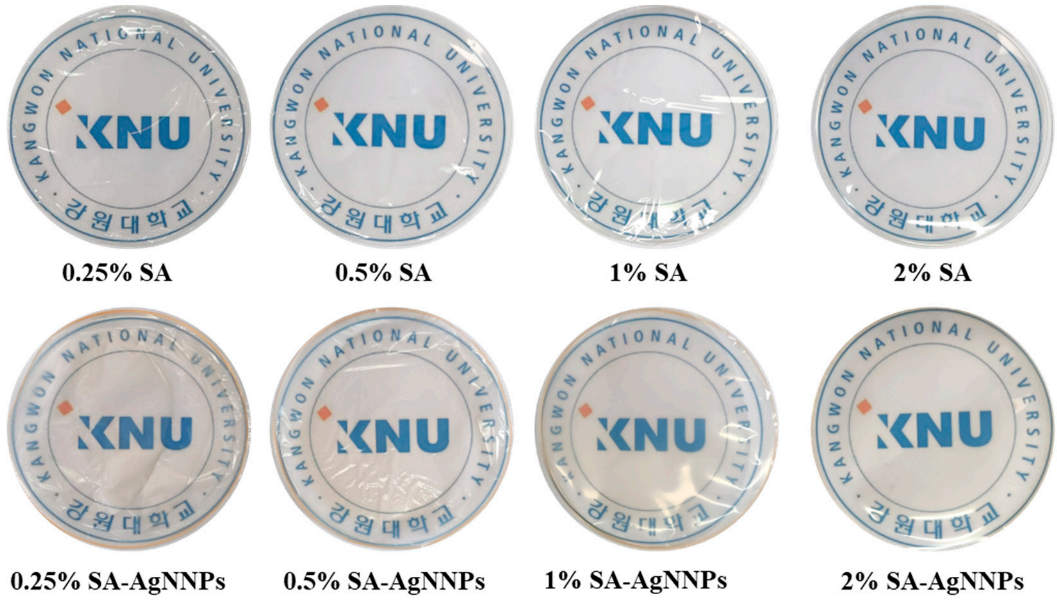

Figure S11. Digital photos of SA films and SA-AgNNPs films with a background of the school logo.

Table S1. Comparative analysis of the antibacterial activity of SA-AgNNPs in well diffusion assays. Where, the concentration of samples used as SA-AgNNPs (50 μg), while TCH (50 μg) alone was used as a positive control.

Samples	Zone of inhibition (mm)				
	B. cereus	*S. aureus*	*L. monocytogenes*	*S. enterica*	*E. coli*
0.25%SA, $AgNO_3$: SNP (2:1)	11.33±0.58	16.67±1.04	14.00±0.87	13.33±0.29	13.17±0.76
0.25%SA, $AgNO_3$: SNP (1:1)	11.17±0.29	17.00±0.5	14.00±0.5	12.83±0.58	13.33±0.58
0.25%SA, $AgNO_3$: SNP (1:2)	9.83±0.29	16.83±0.29	13.50±0.5	11.83±0.58	12.83±0.29
0.5%SA, $AgNO_3$: SNP (2:1)	12±0.5	14.50±0.5	13.83±0.29	13.33±0.29	12.83±0
0.5%SA, $AgNO_3$: SNP (1:1)	8.83±0.5	13.33±0.76	13.33±0.29	13.33±0.29	13.50±0.58
0.5%SA, $AgNO_3$: SNP (1:2)	10.5±0.5	14.00±0.5	13.67±0.58	12.83±0.29	13.33±0.76
1%SA, $AgNO_3$: SNP (2:1)	13.17±0.29	14.17±0.58	14.00±1	14.83±0.29	13.83±0.29
1%SA, $AgNO_3$: SNP (1:1)	13.50±0.50	11.33±2.75	13.83±0.29	13.50±0.50	14.67±0.58
1%SA, $AgNO_3$: SNP (1:2)	13.67±0.76	12.67±2.93	14.67±0.29	14.00±0.50	15.17±0.29
2%SA, $AgNO_3$: SNP (2:1)	13.50±0.5	10.67±2.02	12.83±0.29	12.17±0.29	12.67±0.29
2%SA, $AgNO_3$: SNP (1:1)	11.50±0.5	10.67±2.02	13.50±0	13.50±1	13.00±0.87
2%SA, $AgNO_33$: SNP (1:2)	11.17±0.29	10.17±1.61	13.33±0.76	13.83±1.04	11.67±0.58
TCH	26.67±1.53	25.67±2.52	20.83±0.76	21±1	19.50±0.5
